# Bridging the Gaps in Patient Education for DBS Surgery in Parkinson's Disease

**DOI:** 10.1155/2017/9360354

**Published:** 2017-08-07

**Authors:** Colleen D. Knoop, Robert Kadish, Kathy Hager, Michael C. Park, Paul D. Loprinzi, Kathrin LaFaver

**Affiliations:** ^1^Ochsner Health System, Division of Movement Disorders, 1514 Jefferson Highway, New Orleans, LA 70121, USA; ^2^Department of Neurology, University of Louisville, 220 Abraham Flexner Way, Suite 606, Louisville, KY 40202, USA; ^3^Bellarmine University, 2001 Newburg Road, Miles Hall 301, Louisville, KY 40205, USA; ^4^Department of Neurosurgery and Neurology, University of Minnesota, 420 Delaware Street SE, MMC 96, Minneapolis, MN 55455, USA; ^5^University of Mississippi, 229 Turner Center, Oxford, MS 38677, USA

## Abstract

**Introduction:**

Improvements in quality of life, tremor, and other motor features have been recognized as superior in patients with advanced Parkinson's disease (PD) treated with deep brain stimulation (DBS) surgery versus best medical therapy. We studied a group of patients with PD after undergoing DBS surgery in regard to expectations and satisfaction with DBS outcomes to determine gaps in patient education.

**Methods:**

This study was a retrospective, single academic center chart review and outcome questionnaire sent to patients with PD who had undergone DBS surgery between 2007 and 2014.

**Results:**

All patients surveyed indicated that benefit from DBS surgery met their overall expectations at least partially, but only 46.4% (*SE: 9.6%*) were in complete agreement. 3.6% (*SE: 3.6%*) of participants strongly disagreed that preoperative education prepared them adequately for the procedure and 17.9% (*SE: 7.4%*) only somewhat agreed.

**Conclusions:**

Our findings demonstrate that patients' expectations of DBS surgery in PD were at least partially met. However, there was a considerable percentage of patients who did not feel adequately prepared for the procedure. A structured, multidisciplinary team approach in educating PD patients throughout the different stages of DBS surgery may be helpful in optimizing patients' experience and satisfaction with surgery outcomes.

## 1. Introduction

Parkinson's disease (PD) is a progressive neurological disorder in which the cardinal signs are resting tremor, bradykinesia, rigidity, and loss of postural reflexes [1]. In 2002, the FDA approved deep brain stimulation (DBS) in the subthalamic nucleus (STN) for patients with levodopa-responsive PD [2]. The proposed mechanisms that explain the therapeutic benefit of DBS include local and network-wide electrical and neurochemical effects of stimulation, modulation of oscillatory activity, synaptic plasticity, neuroprotection, and neurogenesis [3–8]. Motor benefits have been documented as long as 10 years after implantation [9].

A good DBS surgical candidate is considered to be a patient with idiopathic PD and good response to levodopa, who is experiencing motor fluctuations, dyskinesias, or refractory tremor despite the best medical therapy and does not suffer from significant cognitive impairment. Prospective DBS candidates need to have an adequate understanding of expected benefits and possible adverse effects from DBS. This is best accomplished through a thorough educational process that spans the pre- to postoperative period. Few studies have been specifically conducted to explore the patient expectations and satisfaction from DBS surgery in relation to the education received by the multidisciplinary treatment team (neurosurgeon, movement disorder specialist, nurses, and neuropsychologists).

Multiple studies have addressed both motor and nonmotor quality-of-life issues after DBS surgery (see Appendix 1 in the Supplementary Material available online at https://doi.org/10.1155/2017/9360354) [10–15]. While motor aspects of PD consistently show improvement with DBS, changes in quality of life (QoL) and mental health are less frequently documented in the literature. Montel and Bungener [12] conducted a study comparing patients undergoing DBS surgery with the best medical therapy alone. The authors found that depression and anxiety were not significantly impacted by the type of therapy received. Those with DBS therapy scored higher in coping techniques, with no particular strategy showing significant differences. The DBS treatment group also experienced decreased QoL measures related to dysarthria.

Ferrara et al. [11] looked at health-related quality of life (HRQoL) and health satisfaction (HS) following DBS surgery. The findings revealed improvements in various HRQoL issues, especially motor function and independence measures. Life satisfaction following DBS did not improve perceived function at work, personal relationships, leisure activities, or living conditions. Social, emotional, and cognitive factors tended to be better predictors of quality of life. Following DBS, energy level and life enjoyment improved significantly. The authors suggested studying HRQoL and HS in subsequent studies, focusing on the enhancement of the patient selection process and consideration of predictive clinical variables.

In a study by Lezcano et al. [13], patients were followed up for five years following DBS surgery. The overall QoL was found to be significantly improved one year after surgery but regressed back to baseline at five years in most measures. Floden et al. [10] retrospectively studied the predictability of QoL measures in 85 patients after STN DBS. They found that QoL improved on 39-item PD questionnaire (PDQ-39) measures for motor function, mood, and self-consciousness but not for speech, cognitive function, and hallucinations. Patients who reported reduced QoL before surgery did not experience a significant increase in QoL after surgery. The authors concluded that DBS increases or preserves QoL in most patients. Hasegawa et al. [16] studied the correlation between patient expectations with satisfaction and outcomes in STN DBS for PD and concluded that pre- and postoperative expectations may play an important role in patient satisfaction and overall success of STN DBS.

The goal of our study was to determine the degree to which patients' expectations from DBS surgery were met postoperatively. Additionally, we sought to gain information that could aid in improving patient education for DBS and creating a patient-centered experience.

## 2. Methods

A retrospective, single academic center study was conducted to evaluate patients' postoperative expectations of DBS. The study was IRB-approved and followed ethical guidelines. A twenty-seven-item questionnaire was developed (Appendix 2 in the Supplementary Material) and administered to patients and a retrospective chart review was performed. Study subjects were identified by using billing codes for PD and DBS from 2007 to 2014. Fifty-two patients were contacted. Patients who had devices removed for any reason were included, regardless of whether they had been reimplanted or not. Initially, patients were recruited via mail. The questionnaire was designed to evaluate patients' expectations, preoperative education, and overall satisfaction with DBS surgery. Most items were evaluated using a Likert scale, but several free response questions were included. Patients' charts were reviewed to identify documentation about DBS education. Additional information gathered included gender, date of birth, education level, ethnicity, age at symptom onset, age at PD diagnosis, age at implant(s), most troublesome symptom(s) prompting DBS, and implanted target area of the brain. Analysis of data was done with STATA, version 12.

## 3. Results

Among the 52 questionnaires mailed, 32 were returned and 29 were included in the analysis, yielding a response rate of 55.8%. One survey was returned unanswered. One subject was excluded from analysis as chart review revealed a diagnosis of essential tremor, rather than PD. The age at DBS surgery ranged from 36 to 86 years with a mean of 66.8 (SD: 10.8) years (Table 1). The majority of patients were males (71%) and the range of disease duration was 2–32 years with a mean of 15.1 (SD: 8.6) years.

The most commonly cited symptoms from the patients' perspective prompting consideration for DBS were tremor (79.3%), dyskinesias (24.1%), and rigidity (13.8%). Another 6.9% of patients reported inadequate on-time and complex medication schedules. Other reasons cited for seeking DBS surgery included walking problems (10.3%), reduced quality of life (10.3%), balance problems (3.4%), freezing of gait (3.4%), and impaired handwriting (3.4%). When participants were asked to identify their sources of DBS education, 96.4% indicated having received information from the provider managing their PD, 60.7% from the neurosurgeon, 46.4% from industry device representatives, 14.3% from nurses or other ancillary staff members, 46.4% from the Internet, and 14.3% from other sources (i.e., support groups, seminars).

**Figure 1:**
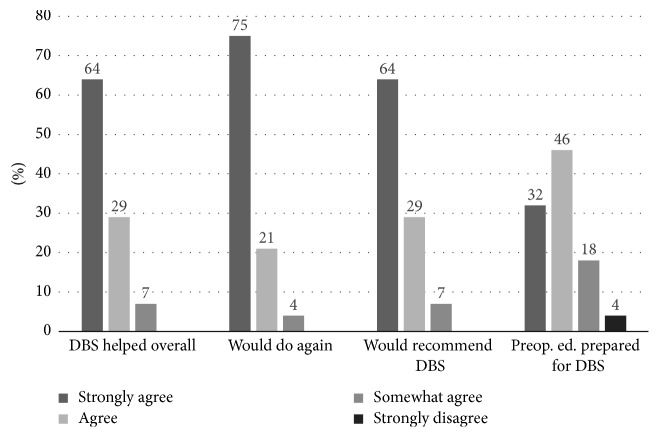
Patient satisfaction with DBS outcomes and preoperative education.

71.4% of the participants reported having been asked about their expectations from DBS prior to surgery; however, a discussion of patient expectations was only documented in medical charts in 48.3%. Postoperatively, 100% of subjects were in at least some agreement that their expectations from DBS surgery were met. More specifically, 46.4% (*SE: 9.6%*) strongly agreed, 39.3% agreed (*SE: 9.4%*), and 14.3% (*SE: 6.7%*) somewhat agreed. Furthermore, 100% of patients surveyed agreed that DBS was overall helpful with 64.3% (*SE: 9.2%*) in strong agreement, 28.6% (*SE: 8.7%*) in agreement, and 7.1% (*SE: 5%*) somewhat in agreement. 100% of participants would elect to undergo DBS surgery again, with 75% (*SE: 8.3%*) in strong agreement, 21.4% (*SE: 7.9%*) in agreement, and 3.6% (*SE: 3.6%*) in some agreement. Similarly, 100% of participants would recommend DBS to someone else with PD, with 64.3% (*SE: 9.2%*) in strong agreement, 28.6% (*SE: 8.7%*) in agreement, and 7.1% (*SE: 5*%) in some agreement. When asked whether preoperative education prepared them adequately about the limitations of DBS, 32.1% (*SE: 8.9%*) strongly agreed, 46.4% (*SE: 9.6%*) agreed, 17.9% (*SE: 7.4%*) somewhat agreed, and 3.6% (*SE: 3.6%*) strongly disagreed (Figure 1).

We also investigated the level to which DBS outcomes met patients' expectations for improvement of various PD symptoms. Expectations were defined as met if the participants strongly agreed, agreed, or somewhat agreed. Overall, patients felt their expectations of symptom improvement were met by DBS (Table 2). Specifically, 82% of patients agreed that their expectations were met for improvement of tremor, rigidity, and dyskinesias, 75% for improvement of bradykinesia, 85% for improvement of “on-time,” and 68% for dystonia.

Table 3 shows data on pre- and postoperative patient expectations across all symptoms and the degree to which expectations were met. Reduction of tremor was identified as the expected outcome by 75% of participants, yet this was only documented in 34.5% of reviewed charts. 79.3% of the participants reported that their expectation for tremor improvement was met. Medication reduction was documented as an expected outcome in 31% of chart reviews, while 21.4% identified this as a desired expectation of DBS on the questionnaire. This expectation was met in 13.8%, whereas 3.4% reported that the expectation was somewhat met, and 3.4% did not have their expectation met. Other patient expectations were felt to be more problematic (C in Table 3), such as improvements in sleep which was cited by 10.3% of participants. In summary, we identified considerable discrepancies in documentations of expected symptom improvements per chart review with patient self-reported expectations on retrospective questionnaire as well as the absence of consistent documentation of patient expectations in medical charts.

## 4. Discussion

The aim of this study was to determine whether patient expectations from DBS surgery in PD were met and to identify gaps in patient education. Data from patient outcome questionnaires showed that a majority of patients (79%) listed tremor as the main reason for pursuing DBS, a symptom that is highly associated with improvement after surgery [17]. Over 96% of the study participants noted that they received DBS education by a PD specialist, but far fewer (61%) recalled having received education from a neurosurgeon. Frequently, patients sought education on their own, with 46% reporting education from Internet sources. While the lower reported rate of education by neurosurgeons may be related to less time spent with the patient throughout the process, it highlights an area for improvement, especially considering the possibility of surgical complications [18]. The large portion of patients receiving information from the Internet highlights the need for providers to guide patients towards reliable sources for information online. A considerable discrepancy between documentation of preoperative patient expectations in charts compared to patient reports of having discussed expectation with providers indicates a need for improvements in documentation of DBS education and following a standard format for this purpose.

Overall, patients had high satisfaction with DBS outcomes and 100% of the participants in our study were in at least partial agreement that their postoperative expectations for DBS surgery were met. Although 96% of the participants were in at least partial agreement when asked whether preoperative education prepared them for DBS surgery, 3.6% strongly disagreed, suggesting a need to optimize the educational process for DBS surgery.

Breit et al. [19] described unmet patient expectations as adverse DBS effects, negatively affecting the stimulation therapy. This is of special concern if the primary patient goals from surgery are not deemed realistic. Family members may also have unrealistic expectations that should be addressed whenever possible. Some patients or families may have unrealistic goals sparked as a result of media depictions of DBS or making generalizations from outcomes observed on other patients [20, 21].

Clinical practice guidelines state that patient education should begin early in the preoperative evaluation process. What can realistically be expected from surgery should be described [17]. Thorough preoperative education should be mandatory, including potential surgical complications [18]. Patients and medical providers should clearly document patient expectations, so that this can be reviewed postoperatively for a more meaningful assessment of goal attainment [22].

As well documented in the literature, most motor symptoms show improvement after DBS surgery. In our sample, 62 to 82% of patients had their expectations of motor-symptom improvements met. Of note, “slowness,” for which 11% of patients disagreed about any improvements postoperatively, is a broad encompassing symptom that may have different meanings. It may be interpreted both in a psychosocial context and in relation to axial signs, which have been documented to relate to dissatisfaction with DBS, especially if present preoperatively [23]. Medication reduction, which often can be accomplished especially after STN-DBS [9, 13, 18, 24], was an expectation that was met for the majority of patients. Expectations for improvement in nonmotor symptoms such as sleep, gait freezing, and handwriting were relatively infrequently mentioned in our survey which likely reflects adequate patient education about the uncertainty of expected benefits from DBS for these symptoms.

Our study has several limitations. This is a retrospective study with a relatively small sample size collected over a seven-year period. Our survey instrument was not evaluated for reliability or validity and was developed as there are currently no established scales to measure patient expectation and satisfaction from DBS surgery. Different practitioners provided the patient with education and documentation in charts was lacking in many instances. There was no protocol in place for a standardized approach to patient education on DBS surgery. The study was performed at a single institution and may not reflect experience with DBS patient education at other centers.

## 5. Conclusions

Despite overall satisfaction in our patient sample with outcomes from DBS, patient expectations should be further explored in a systematic manner. We found considerable discrepancies of documented patient education versus patient reported education on expected symptom improvement. Patient education on DBS should be improved and follow a standardized protocol, ideally involving a multidisciplinary team. Involvement of a nurse educator, a DBS support group, and tailored information over several visits may assist patients in reaching realistic expectations about surgery outcomes and improving their overall satisfaction with DBS surgery. Additional longitudinal studies are needed to further understand the patient-centered experience.

## Supplementary Material

Appendix 1: Literature Review of Patient Satisfaction with DBS Surgery.
Appendix 2: DBS Expectations Questionnaire.

## Figures and Tables

**Table 1:** Patient demographics and clinical characteristics.

Gender	21 M/8 F

Race	100% Caucasian

Disease duration (mean ± SD)	15.1 ± 8.59 years

Age at surgery (mean ± SD)	66.8 ± 10.8 years

Education	43% high school/GED 43% associates degree or higher

DBS targets	STN bilateral 62.1% STN unilateral 17.4% GPi bilateral 13.8% VIM unilateral 6.9%

**Table 2:** Percentage of patients having their motor expectations met after DBS surgery.

Symptom (% of any agreement)	*Strongly agree* *% (SE)*	*Agree* *% (SE)*	*Somewhat agree* *% (SE)*	Neither agree nor disagree % (SE)	Somewhat disagree % (SE)	Disagree % (SE)	Strongly disagree % (SE)	N/A % (SE)
Tremor *N* = 29 (82)	*61 (9.0)*	*14 (7.0)*	*7.0 (5.0)*	0	0	0	0	18 (7.0)
Rigidity *N* = 29 (82)	*32 (9.0)*	*29 (9.0)*	*21 (8.0)*	4.0 (4.0)	4.0 (4.0)	0	0	10 (6.0)
Slowness *N* = 29 (75)	*29 (9.0)*	*21 (8.0)*	*25 (8.0)*	7.0 (5.0)	4.0 (4.0)	7.0 (5.0)	0	7.0 (5.0)
On-time *N* = 28 (85)	*39 (9.0)*	*25 (8.0)*	*21 (8.0)*	11 (1.1)	0	0	0	4.0 (4.0)
Dyskinesia *N* = 29 (82)	*36 (9.0)*	*25 (8.0)*	*21 (8.0)*	7.0 (5.0)	0	0	0	10 (6.0)
Dystonia *N* = 29 (62)	*21 (8.0)*	*29 (9.0)*	*18 (7.0)*	7.0 (5.0)	0	0	0	25 (8.0)

**Table 3:** A comparison of pre- and postoperative patient expectations from DBS surgery. Expectations listed under “A” are deemed realistic expectations with good chances for improvement following DBS surgery. Reducing PD medications (“B”) following DBS surgery is a realistic expectation depending on the target for electrode placement. Symptoms listed under “C” may or may not improve following DBS surgery.

Feature		Preop. expectation documented *N* = 14 % (SE)	Desired expectation for having DBS *N* = 29 % (SE)	Expectation met *N* = 28 % (SE)	Expectation somewhat met *N* = 28 % (SE)	Expectation not met *N* = 28 % (SE)
A	Tremor	35 (9.0)	75 (8.3)	79 (7.7)	3.4 (3.4)	—
	Rigidity	10 (5.8)	8.8 (7.4)	10 (5.8)	—	3.4 (3.4)
	Slowness	—	7.1 (5.0)	3.4 (3.4)	3.4 (3.4)	—
	On-time	21 (7.7)	21 (7.9)	17 (7.1)	3.4 (3.4)	—
	Dyskinesias	17 (7.1)	18 (7.4)	21 (7.7)	3.4 (3.4)	—
	Dystonia	3.4 (3.4)	7.1 (5.0)	3.4 (3.4)	3.4 (3.4)	—

B	Reduce medications	31 (8.7)	21 (7.9)	14 (6.5)	3.4 (3.4)	3.4 (3.4)

C	Sleep	10 (5.8)	3.6 (3.6)	3.4 (3.4)	—	—
	Freezing of gait	6.9 (4.8)	3.6 (3.6)	—	—	—
	Speech	—	3.6 (3.6)	—	—	3.4 (3.4)
	Balance	3.4 (3.4)	3.6 (3.6)	—	—	3.4 (3.4)
	Walking	6.9 (4.8)	14 (6.7)	6.9 (4.8)	—	10 (5.8)
	Writing	—	11 (6.0)	14 (6.5)	—	—
	QoL	—	21 (7.9)	17 (7.1)	—	3.4 (3.4)
	Reduce pain	—	7.1 (5.0)	—	3.4 (3.4)	3.4 (3.4)
	Eat w/utensils	—	3.6 (3.6)	6.9 (4.8)	—	—
	Use tools	—	—	—	3.4 (3.4)	—
	Improve PD	—	11 (6.0)	6.9 (4.8)	3.4 (3.4)	—
	Ride bike	—	3.6 (3.6)	3.4 (3.4)	—	—
	Use of arm	—	3.6 (3.6)	3.4 (3.4)	—	—
	Normal life	—	3.6 (3.6)	3.4 (3.4)	—	—
	Other	24 (8.1)	3.6 (3.6)	3.4 (3.4)	—	—
