# Supplementary material for: Bridging the Gaps in Patient Education for DBS Surgery in Parkinson's Disease
Source: Parkinsons Dis. 2017 Aug 7;2017:9360354. doi: 10.1155/2017/9360354 (PMC5564106; doi:10.1155/2017/9360354)
Supplement: Supplementary file 1 — Appendix 1: Literature Review of Patient Satisfaction with DBS Surgery. Appendix 2: DBS Expectations Questionnaire. [file 9360354.f1.docx]

**Appendix 1: Literature Review of DBS Patient Satisfaction Studies**

| **Author/Year** | **Aim/Purpose** | **Methodology/Sample size** |
| --- | --- | --- |
| **Physician-Rated Outcome Measures** | | |
| Tamma, L., et al. (2003) | Describe pre/post-op UPDRS, up to 1 year | Quantitative, prospective pre/post op comparison ; N=30 (STN) |
| Castrioto, A., et al. (2011) | Describe pre/post-op UPDRS, up to 10 years postop | Quantitative, prospective, pre/post op comparison; N=18 (STN) |
| **Patient-Rated Outcome Measures** | | |
| Törnqvist et al. (2007) | Evaluate results of DBS from the patient’s perspective up to 1 year postop | Qualitative, prospective, pre/post op; N= 16 (8PD, 8ET) (VIM) |
| Maier, F. et al. (2013) | Pre-post op. 3 mo. expectation evaluation of DBS outcomes with negative outcome analysis | Quantitative/qualitative, prospective pre/post op; N=30 (STN) |
| Hasegawa, H. et al. (2014) | Evaluate PD patient expectations pre/post op up to 6 months with PDQ-39 and satisfaction survey | Quantitative/qualitative, prospective, pre/post op; N=22 (STN) |
| Maier, F. et al. (2016) | Compare perceived outcome 1 year after DBS; identify risk factors of dissatisfaction | Quantitative/qualitative, 1 yr postop N=28 (STN) |
| Kubu, C. et al. (2016) | Report prospective data detailing perceived benefit of DBS on commonly cited symptoms and activity goals | Quantitative/qualitative, prospective up to 6 mo. post-op; N=52 |
| **Quality of Life Outcome Measures** | | |
| Montel & Bungener (2009) | Investigate coping measures of PD patients with DBS in regards to QoL, depression, and anxiety 1 year after surgery | Quantitative, post-op matched comparison of PD patients with DBS versus medical therapy only; N=80, (STN) |
| Ferrara, J. et al. (2009) | Provide pilot data about outcomes following DBS using a new, recently validated, DBS health related QoL tool to measure life satisfaction | Quantitative, prospective; N=23 (STN) |
| Floden, D. et al. (2014) | Examine disease, treatment, cognitive, and psychological factors associated with QoL pre/post op and assess predictability of QoL | Quantitative, retrospective; N=85 (STN). |
| Lezcano, E. et al. (2016) | Long term (1, 5 year) impact of DBS on HRQoL and associated factors | Quantitative, prospective; N=69 (STN) |
| Hariz, G. et al. (2016) | Collect and analyze patient’s narratives about everyday experiences of being on chronic DBS | Semi-structures interviews with open ended questions; N=42 (Multiple targets) |
