# Supplementary material for: Bridging the Gaps in Patient Education for DBS Surgery in Parkinson's Disease
Source: Parkinsons Dis. 2017 Aug 7;2017:9360354. doi: 10.1155/2017/9360354 (PMC5564106; doi:10.1155/2017/9360354)
Supplement: Supplementary file 2 [file 9360354.f2.docx]

**Appendix 2: DBS Patient Questionnaire**

**Please fill in the blanks:**

1. What is your current age?

2. What was your age when diagnosed with Parkinson’s disease?

3. How long did you consider Deep Brain Stimulation (DBS) as a treatment

option before having surgery?

4. What year did you first have DBS surgery?

5. If you had surgery on the second side, what year did you have this?

6. What was your most bothersome symptom that made you want to have DBS?

**Please circle the following answers:**

7. What is your gender?

-Male

-Female

8. Which best describes your ethnicity?

-White - Native American or American Indian

-Hispanic or Latino - Asian/ Pacific Islander

-Black or African American -Other

9. What is the highest grade you completed in school?

-Did not attend school -Trade/ technical/ vocational training

-1^st^ to 5^th^ grade -Associate degree

-6^th^ to 8^th^ grade -Bachelor’s degree

-Some high school, no diploma -Master’s degree

-High school graduate or equivalent -Doctorate degree

-Some college, no degree

**Please check all that apply:**

10. Who provided your education about DBS surgery?

______ Provider that treated your Parkinson’s disease (physician,

nurse practitioner, physician assistant)

______ Neurosurgeon

______ Medtronic Educational Tools (i.e. Videos, DVDs, brochures)

______ Nurse or other staff member

______ Internet

______ Other (i.e. support group, seminars, TV)

______No one educated me about DBS surgery

11. Using the following scale, please rate how helpful each of the following

sources were in providing your education before surgery? If no one educated

you, please skip this question.

1. Most helpful
2. Very helpful
3. Helpful
4. Somewhat helpful
5. Not helpful
6. Did not receive education from this source

______ Provider that treated your Parkinson’s disease (physician,

nurse practitioner, physician assistant)

______ Neurosurgeon

______ Medtronic Educational Tools (i.e. Videos, DVDs, brochures)

______ Nurse or other staff member

______ Internet

______ Other (i.e. support group, seminars, TV)

**Please circle the following answers:**

12. Before you had DBS surgery, did anyone ask you what your expectations were?

-Yes

-No

13. Did you have DBS surgery with the University of Louisville Movement Disorder program?

-Yes

-No

**On the following questions, please circle the ONE answer that best describes your level of agreement:**

14. DBS met my expectation for improvement of tremor?

[1] Strongly agree

[2] Agree

[3] Somewhat agree

[4] Neither agree or disagree

[5] Somewhat disagree

[6] Disagree

[7] Strongly disagree

[8] N/A- I did not have DBS for tremor.

15. DBS met my expectation for improvement of stiffness/rigidity.

[1] Strongly agree

[2] Agree

[3] Somewhat agree

[4] Neither agree or disagree

[5] Somewhat disagree

[6] Disagree

[7] Strongly disagree

[8] N/A- I did not have DBS for stiffness/rigidity.

**On the following questions, please circle the ONE answer that best describes your level of agreement:**

16. DBS met my expectation for improvement in slowness of movement.

[1] Strongly agree

[2] Agree

[3] Somewhat agree

[4] Neither agree or disagree

[5] Somewhat disagree

[6] Disagree

[7] Strongly disagree

[8] N/A- I did not have DBS for slowness of movement.

17. DBS met my expectation for improving on/off time (the time when you feel

your symptoms are best controlled)?

[1] Strongly agree

[2] Agree

[3] Somewhat agree

[4] Neither agree or disagree

[5] Somewhat disagree

[6] Disagree

[7] Strongly disagree

[8] N/A- I did not have DBS for improvement of on/off time.

**On the following questions, please circle the ONE answer that best describes your level of agreement:**

18. DBS met my expectation for improving dyskinesias (dancing, wiggling, or

writhing movements)?

[1] Strongly agree

[2] Agree

[3] Somewhat agree

[4] Neither agree or disagree

[5] Somewhat disagree

[6] Disagree

[7] Strongly disagree

[8] N/A- I did not have DBS for dyskinesias.

19. DBS met my expectation of improving dystonia (muscle pulling, cramping)?

[1] Strongly agree

[2] Agree

[3] Somewhat agree

[4] Neither agree or disagree

[5] Somewhat disagree

[6] Disagree

[7] Strongly disagree

[8] N/A- I did not have DBS for dystonia

**On the following questions, please circle the ONE answer that best describes your level of agreement:**

20. DBS has helped me overall.

[1] Strongly agree

[2] Agree

[3] Somewhat agree

[4] Neither agree or disagree

[5] Somewhat disagree

[6] Disagree

[7] Strongly disagree

21. If I had it to do all over again, I would still have DBS.

[1] Strongly agree

[2] Agree

[3] Somewhat agree

[4] Neither agree or disagree

[5] Somewhat disagree

[6] Disagree

[7] Strongly disagree

**On the following questions, please circle the ONE answer that best describes your level of agreement:**

22. I would recommend DBS to others living with Parkinson’s disease.

[1] Strongly agree

[2] Agree

[3] Somewhat agree

[4] Neither agree or disagree

[5] Somewhat disagree

[6] Disagree

[7] Strongly disagree

23. DBS met my overall expectations.

[1] Strongly agree

[2] Agree

[3] Somewhat agree

[4] Neither agree or disagree

[5] Somewhat disagree

[6] Disagree

[7] Strongly disagree

**On the following questions, please circle the ONE answer that best describes your level of agreement:**

24. The education I received before surgery prepared me for what this device does

and does not improve.

[1] Strongly agree

[2] Agree

[3] Somewhat agree

[4] Neither agree or disagree

[5] Somewhat disagree

[6] Disagree

[7] Strongly disagree

[8] N/A- I did not have DBS for this symptom

**Please answer in your own words:**

25. What did you expect DBS to do for you?

26. Which of these expectations were met by DBS?

27. Now that you have had DBS, is there additional information that you wish had

been given before you had surgery?
